# Study on the Mechanical Properties of Door-Shaped Rebar of a CRTS III Slab Track Under Temperature Load

**DOI:** 10.3390/ma18071612

**Published:** 2025-04-02

**Authors:** Marui Han, Zhiping Zeng, Qiuyi Li, Peicheng Li, Wei Wei, Weidong Wang, Abdulmumin Ahmed Shuaibu

**Affiliations:** 1 School of Civil Engineering, Central South University, Changsha 410075, China; 2 MOE Key Laboratory of Engineering Structures of Heavy Haul Railway (Central South University), Changsha 410075, China; 3 China Railway Fourth Survey and Design Institute Group Co., Ltd., Wuhan 430063, China; 4 China Railway Eryuan Engineering Group Co., Ltd., Chengdu 610031, China; 5 Department of Civil Engineering, Faculty of Engineering, Ahmadu Bello University, Zaria 800242, Nigeria

**Keywords:** CRTS III slab track, door-shaped rebar, damage, temperature gradient

## Abstract

CRTS III (China Railway Track System) slab track is a typical multi-layer composite structure. Door-shaped rebar is an important connecting component. Studying its service characteristics is crucial for disease prevention and control. To investigate the mechanical properties of door-shaped rebar under multi-factor coupling, a three-dimensional finite element model was developed and validated with full-scale tests. Then, considering the initial defects of self-compacting concrete (SCC), interlayer cracks and other damages, the mechanical properties of door-shaped rebar of a CRTS III slab track under temperature load were studied. The results show that under the action of an extreme temperature gradient, the maximum vertical displacement of the track slab occurs in the middle and corner positions of the slab with the positive temperature gradient, with displacement values of 0.87 mm and -0.99 mm respectively. Under positive and negative temperature gradients, the stress of the door-shaped rebar at the interface between the track slab and the SCC layer is 12.9 MPa and 16.5 MPa, respectively. When considering the conditions of the SCC slurry layer, the stress of the door-shaped rebar in the SCC layer under the action of a temperature gradient is generally in an increasing state. When there is a crack at the interface of the SCC layer, the stress of the door-shaped rebar unit in the crack area changes significantly, and the crack at the edge of track slab has the greatest impact on the stress of the door-shaped rebar under the positive temperature gradient, which is 157.9 MPa. The research results provide a theoretical basis for disease control.

## 1. Introduction

The CRTS III slab ballastless track is a new type of track structure system independently developed by China based on the advantages of various track structures [1]. Due to its significant economic and technical advantages in railway track construction, it has been widely used at home and abroad. The CRTS III slab track structure adopts the design concept of “coordinated force, coordinated deformation, and coordinated vibration” [2]. Self-compacting concrete (SCC) is cast on site as the filling layer under the track slab, and door-shaped connecting steel bars are set between the track slab and the SCC layer to form a tightly connected “composite slab” structure. Affected by meteorological factors, such as the solar radiation intensity, temperature and wind speed, the track structure will produce warping stress under the action of a temperature gradient, which will cause warping deformation [3,4]. The door-shaped rebar plays an important role in coordinated deformation and stress between the track slab and the SCC layer.

The CRTS III slab track structure is composed of multiple reinforced concrete structural layers. Since the track slab is a prefabricated slab and the SCC layer is cast on site on the lower layer of the track slab, the interface is the bonding surface between the new and old concrete, which may have problems, such as uneven bonding or potential local defects [5,6]. The two layers are connected by door-shaped rebar to form a special composite track structure that bears the uniform force. In service, under the influence of factors, such as the train load, temperature, rain and uneven settlement of the foundation under the track, structural damage gradually occurs through long-term accumulation. To a certain extent, separation may occur at the interface between the track slab and the SCC layer, affecting the normal use of the track structure, reducing riding comfort, and also posing a huge threat to driving safety [7,8,9].

The temperature load, as one of the important factors affecting the mechanical properties of a ballastless track, has attracted the attention of many scholars. To investigate the temperature characteristics of a CRTS III ballastless track structure in a natural environment, Hubing L et al. conducted a full-scale temperature field test on this structure. Drawing on theories from meteorology, heat transfer and radiation, a unified and practical method for analyzing temperature fields under outdoor conditions was established, along with an assessment of the impact of these temperature variations on the CRTS III track structure [10]. Li Z et al. focused on the temperature deformation of the CRTS III track on the viaduct and the changes in the vehicle–track–beam dynamic response caused by the deformation [11]. Ran L et al. used the sequential coupling method to calculate the warping deformation of the CRTS III track slab under the combined effects of the temperature field, shrinkage strain field and prestressed tendons [12]. Tao S et al. proposed a method combining finite element analysis and machine learning to predict the vertical temperature gradient of a ballastless track caused by solar radiation [13,14]. Paulo AG Piloto et al. verified the CNU model for simulating the track temperature through experimental data and developed a Python library (railtemp) with slightly better performance. In addition, they also proposed a method for determining the track temperature based on the air temperature and a method for calculating the temperature of steel under fire [15,16]. These research results can provide a reliable reference for the reasonable selection of temperature load in this paper.

The CRTS III slab track has been in large-scale use for more than ten years, and research on its service performance and damage has been carried out. Zhiping Z et al. studied its long-term service performance under fatigue load through indoor tests and a three-dimensional finite element model analysis [17,18,19]. Bin Y et al. studied the influence of interface cracks on the dynamic response of the CRTS III ballastless track system on the bridge [20]. Qingyuan X et al. established a high-speed train–CRTS III Slab track-subgrade coupled dynamic model and theoretically studied and analyzed the influence of the number of vehicles on the dynamic characteristics of the high-speed train–CRTS III Slab track-subgrade coupled system under the smooth and random track irregularities of ordinary and damped CRTS III tracks [21]. Zhang Y et al. proposed a refined fatigue cohesive zone model considering the factors of stiffness, strength and energy fracture degradation to study the interface damage of the CRTS III slab track under the combined effects of temperature and mixed passenger and freight train loads and proved the inhibitory effect of door-shaped rebar on the development of interface damage [22].

It is not difficult to find that the above studies on the service performance of the CRTS III slab track under temperature loads have limited research on door-shaped rebar. As an important connecting component, the door-shaped rebar is very important for the coordinated work of the structural system. Furthermore, the study on the properties of door-shaped rebar under the coupling of various initial defects and damage is not in-depth enough. Therefore, this paper considers the initial defects of SCC, interlayer cracks and other damages and studies the mechanical properties of CRTS III slab track door-shaped rebar under a temperature load. Firstly, a three-dimensional finite element analysis model was established and compared with the indoor full-scale test results to verify the reliability of the model. Then, the properties of the door-shaped rebar under extreme temperature gradients under different working conditions were determined.

## 2. Model Establishment and Experimental Verification

### 2.1. Finite Element Calculation Model of CRTS III Slab Ballastless Track

**Figure 1:**
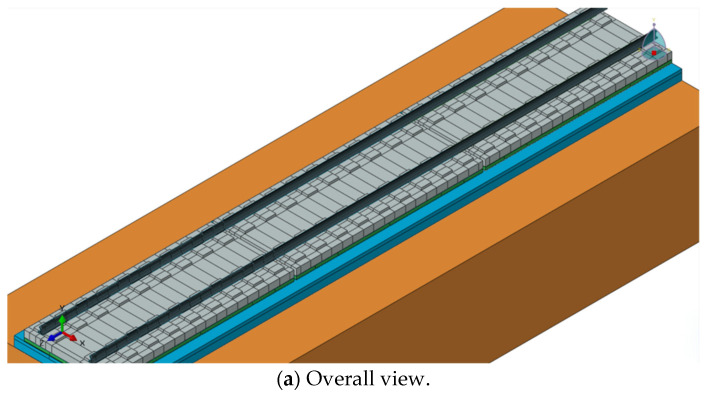

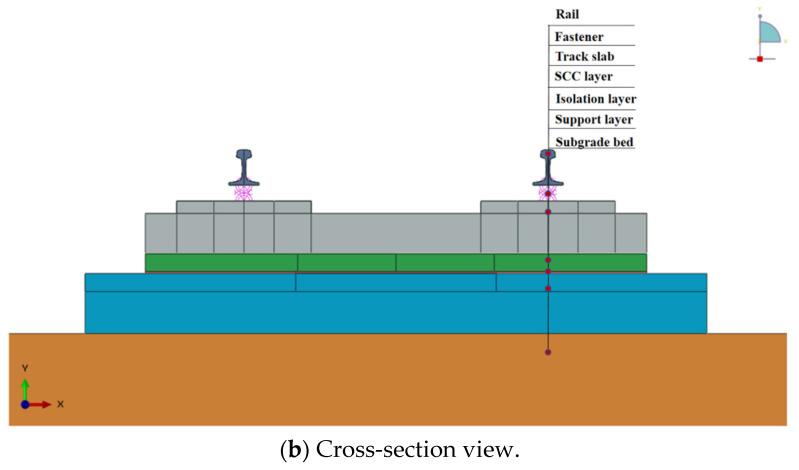
Three-dimensional finite element model of CRTS III slab track structure on subgrade.

In order to be consistent with the actual situation and to facilitate a comparative verification by keeping consistent with the indoor test conditions, a three-dimensional finite element model of the CRTS III slab track structure on the subgrade was established. The modeling software used is the 2021 version of ABAQUS. The calculation model takes into account structures, such as rails, fasteners, precast track slabs, door-shaped rebar, the post-cast SCC layer, the isolation layer and the hydraulic support layer. This paper establishes a model containing three track slabs and takes the middle slab as the main research object, as shown in Figure 1.

**Figure 2:**
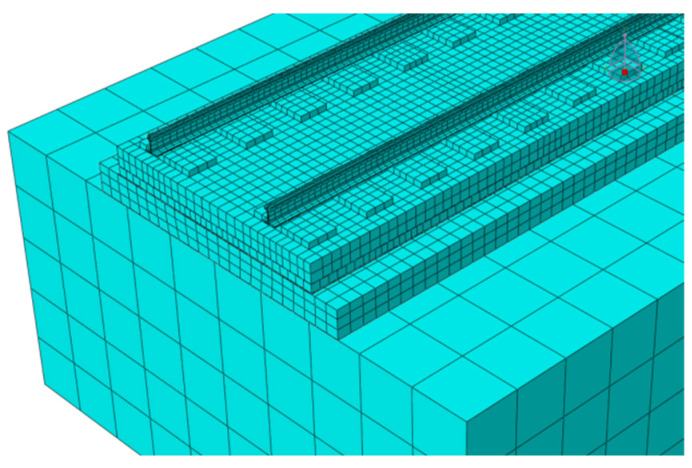
Meshing situation.

**Figure 3:**
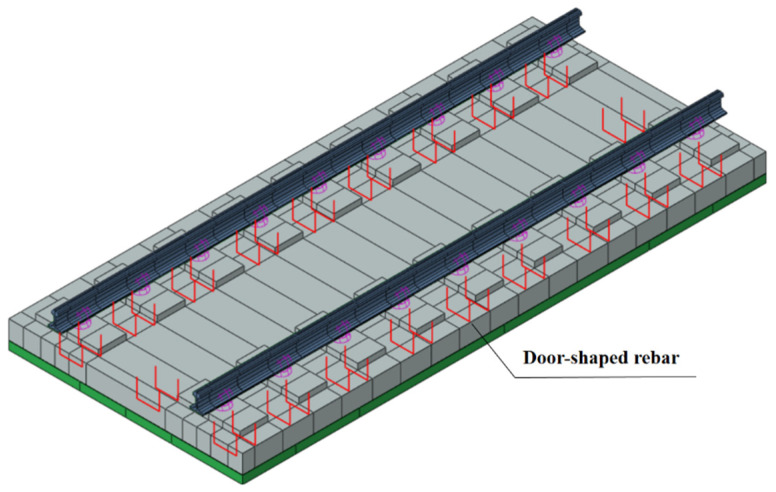
Door-shaped rebar arrangement and characterization.

In the model, except for fasteners and steel bars, all parts are modeled using solid elements C3D8R.The fastener is simulated by the spring-damper unit CONN3D2, and the bottom surface of the rail and the sleeper are connected after point-to-surface coupling according to the actual contact area. The mesh division is shown in Figure 2. The door-shaped rebar is simulated by truss units, the upper part of which is pre-installed and consolidated in the track slab in the factory, and the lower part is wrapped after pouring SCC on site. Therefore, the interaction relationship is characterized in the finite element model through built-in embedding, as shown in Figure 3. The connection between the track slab and the SCC is simulated via binding. The SCC, isolation layer, support layer and subgrade are all in surface contact, with hard contact in the normal direction and friction contact in the tangential direction. The friction coefficient between the supporting layer and the subgrade is 0.4, and the rest is 0.8 [23]. The model side is symmetrically constrained, and the bottom is fixed.

### 2.2. Model Parameters

The rail is of the 60 kg/m standard. The fasteners use the WJ-8 fastener system. The vertical stiffness of the fasteners is 35 kN/mm, the damping is 0.05 kN·s/mm [24] and the spacing is 0.63 m. The track slab is a prefabricated prestressed reinforced concrete structure using C60 concrete. A single track slab is 5.6 m long, 2.5 m wide and 0.2 m thick. Each slab is laid with 9 sets of fasteners, and the slab seam is 0.07 m. The SCC layer is 2.5 m wide and 0.1 m thick. It uses C40 concrete. The longitudinal length is 5.6 m, the same as the track slab. The seam width is 0.07 m, and a boss is set on the lower surface to connect it to the base slab. The supporting layer is a hydraulic mixture supporting layer, which is C20 concrete, 3.1 m wide and 0.3 m thick, and is laid continuously along the longitudinal direction of the line. The foundation bed is 3 m thick, with the surface layer having a thickness of 0.4 m and the bottom layer having a thickness of 2.6 m. The material parameters of each component are shown in Table 1 [2].

### 2.3. Experimental Verification of Model Calculation Reliability

#### 2.3.1. Indoor Full-Scale Static Load Test

**Figure 4:**
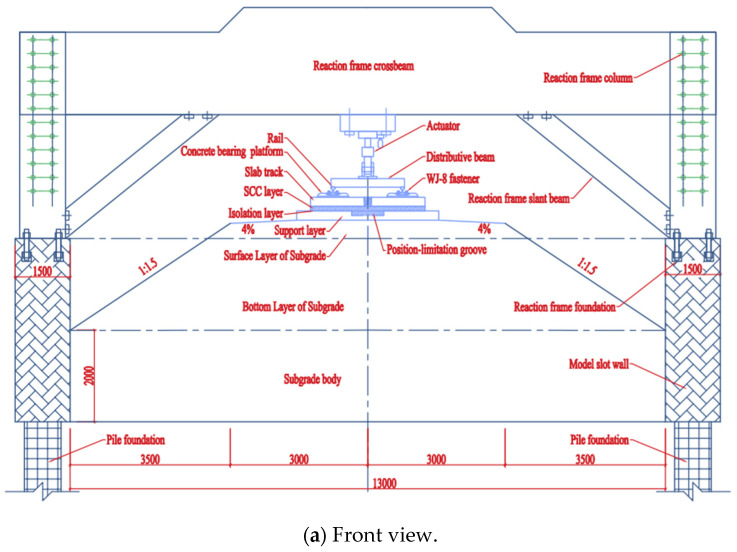

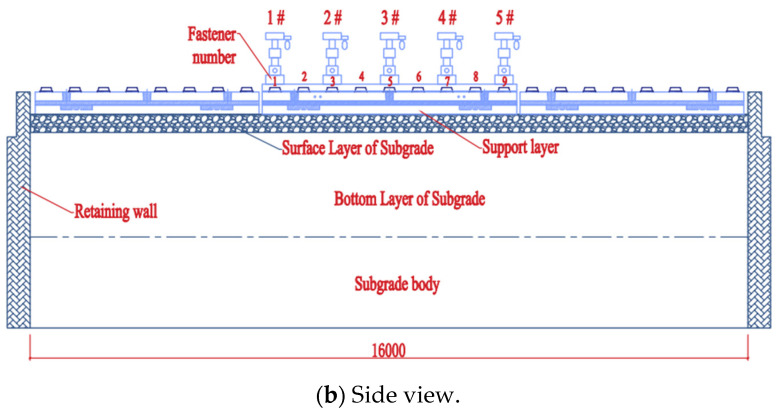
Test system.

**Figure 5:**
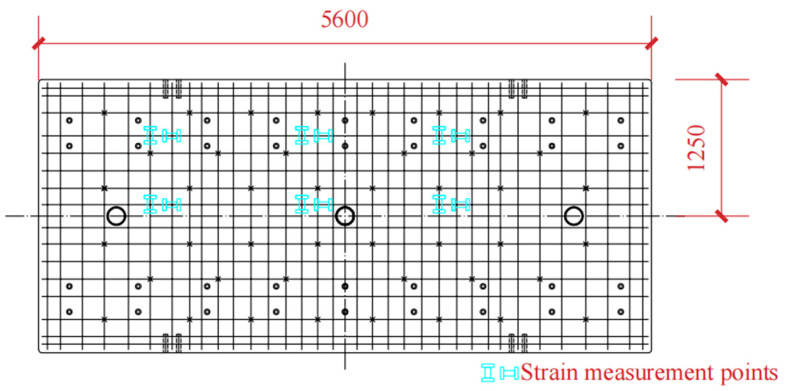
Strain gauge placement.

Our research team designed and produced a full-scale indoor test model of the CRTS III slab track-subgrade. All components were manufactured in strict accordance with the design and process requirements, striving to be consistent with the on-site laying conditions [17]. The test model consists of three track slabs, with the middle slab as the main research object. The test system is shown in Figure 4. During the construction process, strain test elements are embedded in the corresponding positions of each layer of the track structure, and displacement sensors are installed after pouring is completed [25,26]. The strain test includes the longitudinal and transverse strain of track slab concrete, the longitudinal and transverse strain of SCC, and the longitudinal and transverse strain of the base. The strain gauge layout is shown in Figure 5. The displacement test includes two kinds of relative displacement. One is between the rail and the track plate, and the other is between the track plate and the base. The sensors are arranged at the No. 1, 5 and 9 fasteners (see Figure 4b for numbers). The strain measurement uses a vibration strain gauge equipped with a temperature compensation function with an accuracy of ±0.1 με; the displacement measurement uses a micrometer with an accuracy of ±0.001 m. Each test element was calibrated and checked before the test.

The first part of the test content is to apply static loads in stages to the full-scale model of the CRTS III slab track-subgrade under different fatigue states and observe the changes in deformation, strain, etc., in different parts. Before the test, apply a preload of no more than 20% of the upper limit load 1 to 2 times to eliminate looseness and poor contact, press the components firmly and ensure the normal operation of the instrument. In the formal test, a graded static load test was first performed, with graded loading increments of 0, 10, 30, 50, 70, 90, 110 and 130 kN. Subsequently, the machine is shut down for static load tests after 80, 200, 600, 1000, 1600, 2000 and 30 million fatigue load actions, respectively, with the same loading gradient.

The second part of the test is to apply a temperature gradient load to the track structure in an environmental chamber. The negative temperature gradient values are −15, −20, −25, −30, −35 and −35 °C/m respectively, and the positive temperature gradient values are 50, 65, 75, 85 and 95 °C/m, respectively.

Each working condition was repeated three times to meet the repeatability requirement and reduce the influence of abnormal conditions.

#### 2.3.2. Comparative Verification

**Figure 6:**
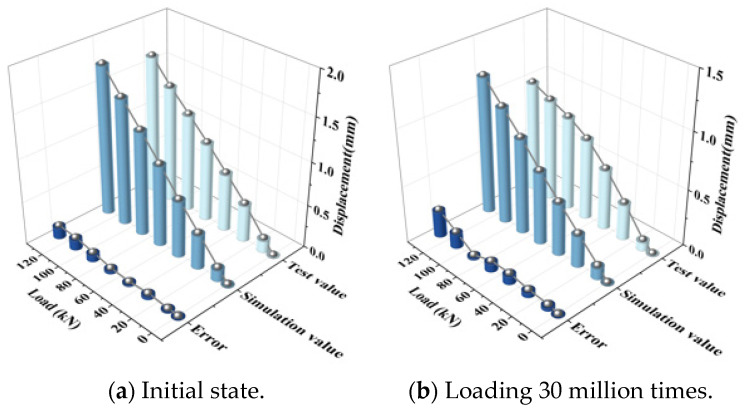
Displacement of rail relative to track slab.

**Figure 7:**
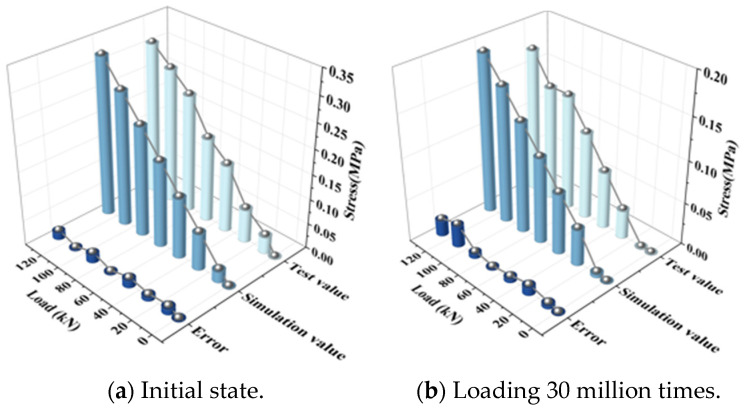
Strain of track slab.

For fatigue tests, the test results for graded loading at the initial state and after 30 million fatigue load cycles are compared with the finite element analysis results. The stress is obtained based on the calculation relationship between stress and strain. The fatigue state in the finite element model is characterized by the change in stiffness of each component, and the values can be found in reference [17]. The displacement and stress comparison of each part is shown in Figure 6 and Figure 7. The comparison of test and model data under different fatigue conditions can prove that the model calculation is correct under different service conditions.

**Figure 8:**
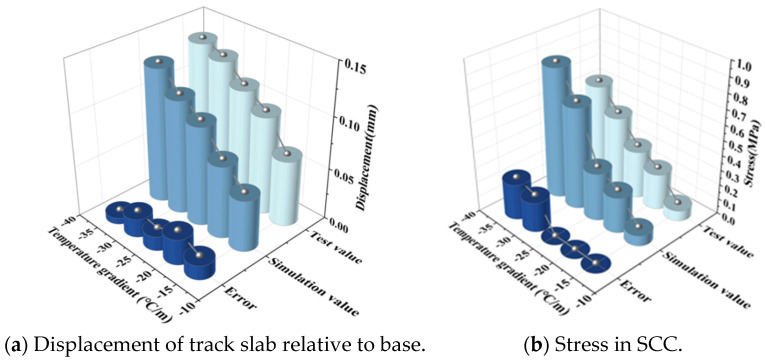
Negative temperature gradient.

**Figure 9:**
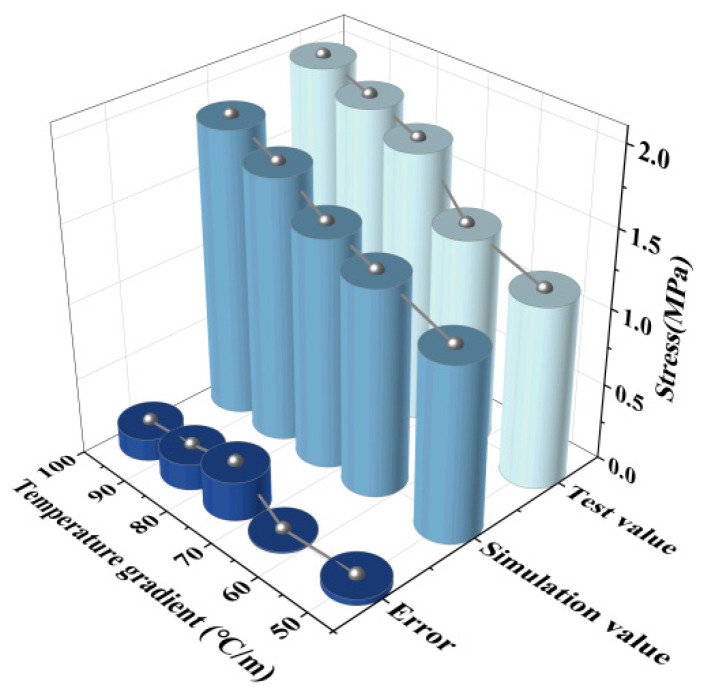
Positive temperature gradient.

The temperature load test selects the relative displacement of the track slab to the base and the stress of SCC to compare it with the finite element calculation results. The comparisons of the results are shown in Figure 8 and Figure 9.

It can be seen that the finite element model in this paper is reliable in the static calculation. In addition, Section 3.2 further verifies the accuracy of the model for the mechanical analysis under temperature loads.

## 3. Analysis of Mechanical Characteristics of Track Structure Under Temperature Load

### 3.1. Load Parameters

According to the Code for Design of High Speed Railway [27], the maximum positive temperature gradient of a ballastless track is 90 °C/m, the maximum negative temperature gradient is 45 °C/m and the commonly used temperature gradient is 1/2 of the maximum value. China’s high-speed railways cover a vast area with large climate differences. In actual conditions, the temperature gradient is not a fixed value. According to the measurement results of reference [28], there are certain differences in the temperature gradient of the track structure under different regions and environmental conditions, but the maximum positive and negative temperature gradients are basically within the maximum gradient range taken by the specification, and the time when the positive and negative temperature gradients appear has a certain regularity. The maximum daily positive temperature gradient appears between 14:00 and 15:00, and the maximum negative temperature gradient of the day generally occurs between 5:00 and 8:00. Moreover, the temperature gradient at different times presents a nonlinear distribution in a certain state.

This section mainly studies the mechanical characteristics of the CRTS III slab track structure under extreme temperature gradient loads. Both positive and negative temperature gradients are taken as the maximum value according to the specification. Since the negative temperature gradient varies nonlinearly along the depth of the track structure and the SCC has almost no gradient change, the negative temperature gradient only acts on the track slab. The deadweight of the track structure is taken into account in the calculations.

### 3.2. Analysis of Vertical Displacement Changes in Track Structure

**Figure 10:**
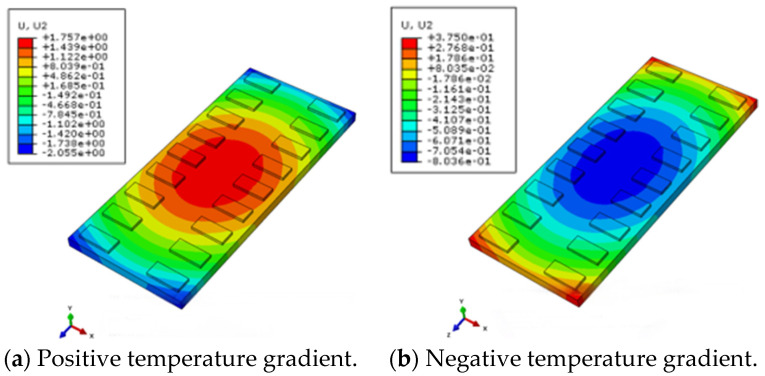
Vertical displacement of track slab under temperature load.

**Figure 11:**
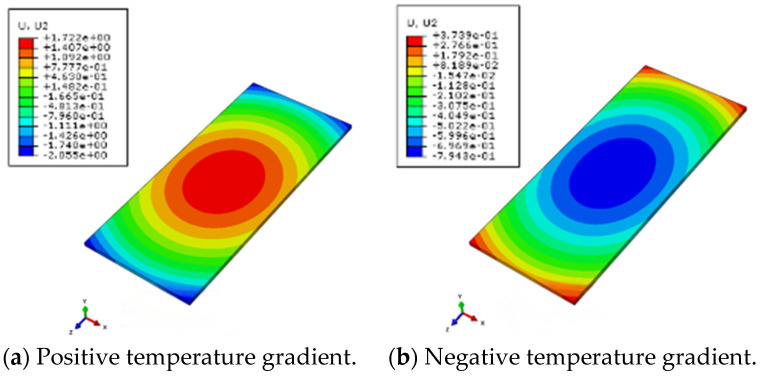
Vertical displacement of SCC layer under temperature load.

Under the action of positive or negative temperature gradients, the track structure will experience deformation, such as camber in the middle of the track slab or warping at the corner of the track slab [29]. Figure 10 and Figure 11 are the vertical displacement changes of the track slab and the SCC layer under a temperature load. The results agree well with the actual deformation results of the track structure.

**Figure 12:**
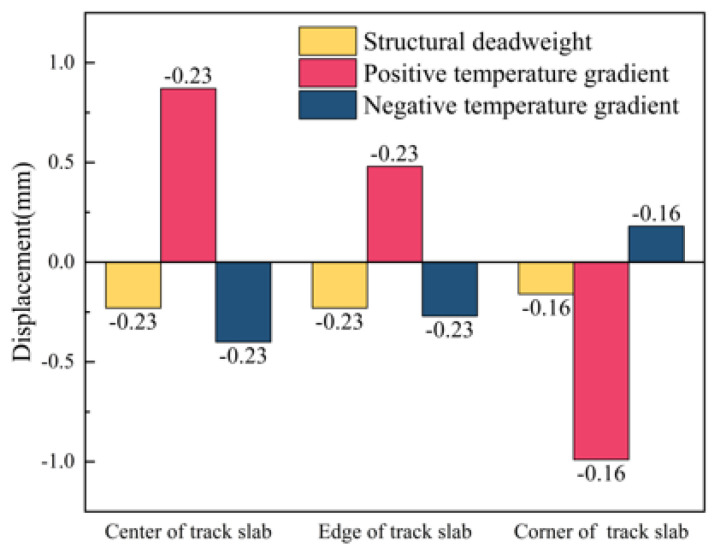
Vertical displacement of track slab at different positions under a temperature load.

The vertical displacement of the track slab at different positions under the action of the structure’s deadweight and positive and negative temperature gradients is shown in Figure 12. The center of the track slab is the intersection of the longitudinal and transverse center lines of the track structure, the edge of the track slab is the middle edge of the track structure and the corner of the track slab is the end corner of the track slab. Displacement values are negative when they are vertically downward. Under the action of its own weight, the structure has a certain amount of the initial displacement value. Under the action of positive and negative temperature gradients, the maximum vertical displacements appear in the center and corner of the track slab under the positive temperature gradient, respectively, with displacement values of 0.87 mm and −0.99 mm.

### 3.3. Analysis of Stress Changes of Door-Shaped Rebar

**Figure 13:**
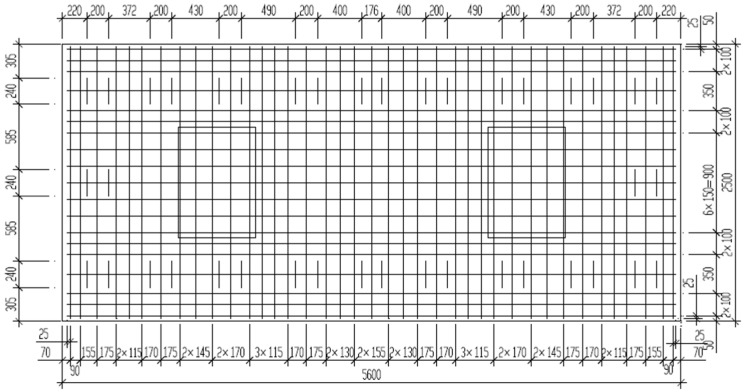
Track slab planar reinforcement diagram.

The door-shaped rebar is a “U”-shaped steel bar with a vertical height of 200 mm and a horizontal length of 240 mm connecting the track slab and the SCC layer. The height of the vertical part extending into the track slab is 150 mm, and the height in the SCC layer is 50 mm. The plane position arrangement of the door-shaped rebar is shown in Figure 13.

**Figure 14:**
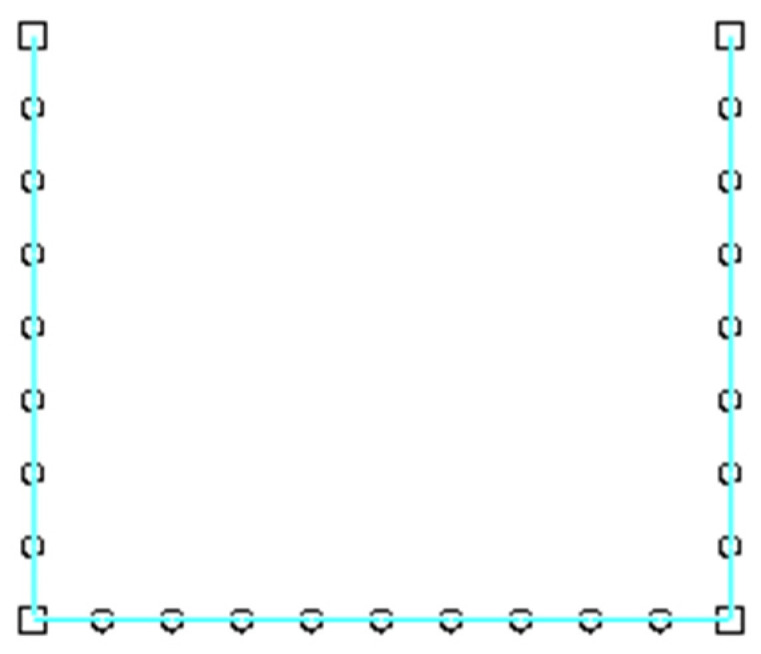
Door-shaped rebar unit and node division.

In the finite element calculation, the unit division of the door-shaped rebar is shown in Figure 14. There are 8 units in the vertical direction, and the unit length is 25 mm; there are 10 units in the horizontal direction, and the unit length is 24 mm. The units of the door-shaped rebar from bottom to top in the vertical direction are numbered 1–8, among which units 1 and 2 are located in the SCC layer; nodes 3–8 are located in the track slab.

**Figure 15:**
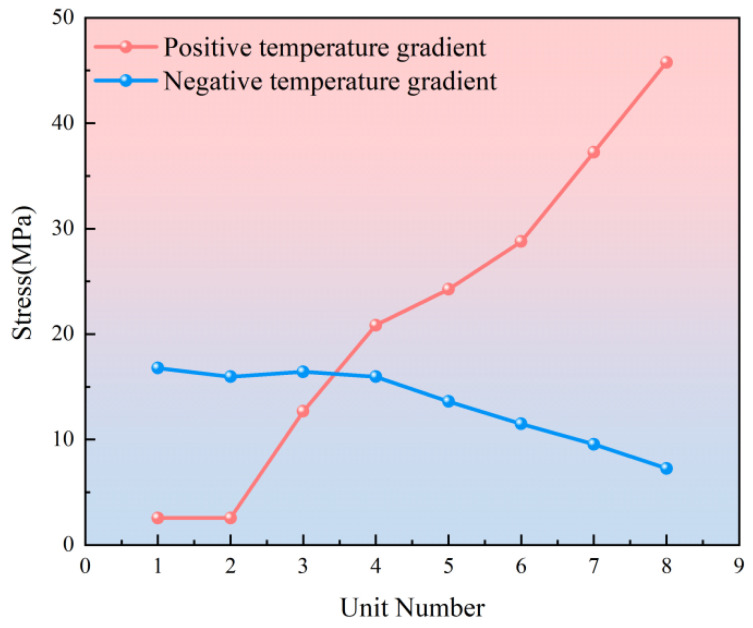
Stress distribution diagram of door-shaped rebar under s temperature gradient.

Under the action of a positive temperature gradient, the stress of the door-shaped rebar increases vertically from bottom to top, and the stress at the top of the door-shaped rebar is the largest. The stress situation under a negative temperature gradient is opposite, as shown in Figure 15. The maximum stress of the door-shaped rebar under the positive temperature gradient is 45.8 MPa, and the maximum stress under the negative temperature gradient is 16.8 MPa. Under positive and negative temperature gradients, the stress of the door-shaped rebar at the interface between the track slab and the SCC layer is 12.9 MPa and 16.5 MPa, respectively.

## 4. Stress Distribution of Door-Shaped Rebar When Defects Exist in SCC

The SCC has the characteristics of high fluidity, no segregation and no water seepage. It can be filled into every corner of the formwork by its own gravity without vibration, and the quality is uniform after filling. In the actual construction process, due to factors such as the objective environment or technical level, it is inevitable that quality defects may occur. This section mainly analyzes the stress distribution law of the door-shaped rebar under the state of bond failure between the laitance layer and the door-shaped rebar after the laitance layer appears in the SCC.

There is no quantitative analysis standard for the identification of the floating slurry layer, and it is mainly based on subjective observations. Reference [29] attempted to propose a method for testing the laitance of concrete to characterize the laitance of concrete. The test results in the literature show that the average thickness of the slurry layer for fresh concrete is between 8 and 20 mm. In this section, 20 mm is selected as the maximum thickness of the slurry layer of SCC, and the influence of slurry layers of different thicknesses on the stress of door-shaped rebar under a temperature load is considered, respectively.

**Figure 16:**
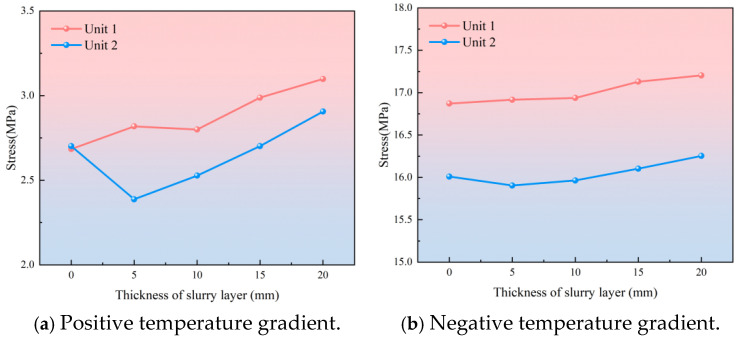
Stress distribution of door-shaped rebar.

The calculation model uses the form of “life and death unit” to make the bond between the SCC layer and the door-shaped rebar fail at a certain thickness, thereby simulating different laitance layer thicknesses. The calculation results show that under the action of the temperature load, the slurry layer has a weak effect on the stress level of the door-shaped rebar in the track slab but has a greater effect on the stress change of the door-shaped rebar in the SCC layer. The stress changes of door-shaped rebar unit 1 and unit 2 in the SCC layer under positive and negative temperature gradients are shown in Figure 16. It can be seen from the figure that the unit stress in the SCC layer under the action of the temperature gradient is generally in an increasing state. Among them, the stress of unit 2 under the positive temperature gradient has a trend of first decreasing and then increasing, and the overall increase is not significant. The increase rates of positive temperature gradients are 15.1% and 9.7%, respectively, and the increase rates of negative temperature gradients are 2.0% and 1.6%, respectively.

## 5. Stress Distribution Law of Door-Shaped Rebar Under Interface Separation Condition

The initial defects in the SCC layer are important factors for the initiation of interlayer cracks [30,31]. In this section, interlayer separation is divided into slab edge separation, the separation under the rail, slab middle separation and complete interface separation under extreme conditions according to their locations for analysis. For all separation conditions, the floating slurry layer is 20 mm.

### 5.1. Stress Distribution of Door-Shaped Rebar Under the Track Slab Edge Gap

**Figure 17:**
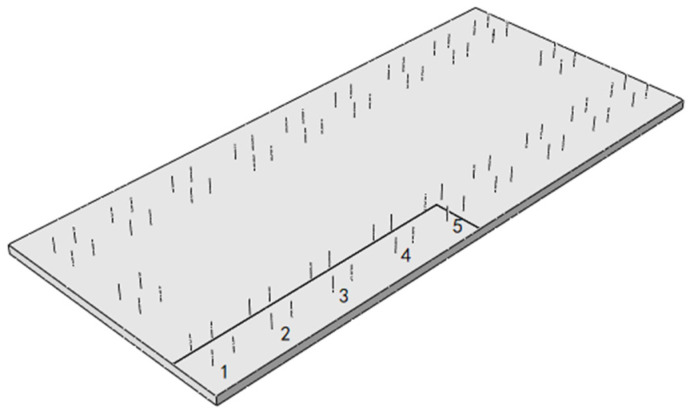
SCC slab edge gap.

The slab edge gap is taken from the SCC layer with a gap width of 500 mm and a height of 2 mm. The gap starts from the slab corner and develops longitudinally along the line to the middle of the slab. The relative position of the gap area and the door-shaped rebar is shown in Figure 17. It can be seen from the figure that there are five groups of door-shaped rebars located within the gap range, which are numbered 1–5 from the end of the track slab to the middle of the track slab. Based on this, the vertical stress distribution of the door-shaped rebar in this state is analyzed, and the stress symbol uses “tension is positive and compression is negative” to indicate the stress direction.

**Figure 18:**
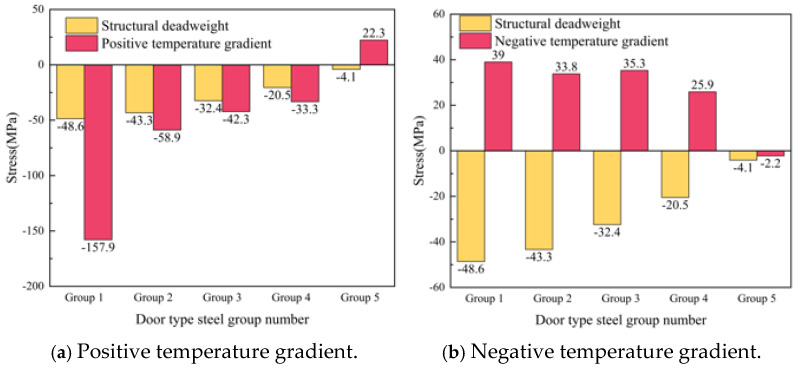
Stress of the door-shaped rebar in the track slab edge separation area.

The calculation results show that when the track structure is intact, the stress on the door-shaped rebar is small and can be ignored only under the action of the track structure’s own weight. When a gap appears on the edge of the track slab, the stress of the door-shaped rebar unit in the gap area changes significantly under the action of the deadweight of the track structure and the temperature load. The stresses of the five groups of door-shaped rebars at the gap interface under the action of deadweight and after the application of positive and negative temperature loads are shown in Figure 18. Under the action of the deadweight of the structure, the compressive stress of the door-shaped rebar in the gap area increased significantly. The stress of the door-shaped rebar in the first group (slab corner position) was the largest, which was 48.6 MPa. The stress of the door-shaped rebar in the fifth group (slab middle position) was 4.1 MPa, which was 8.4% of the maximum value. The stress of the door-shaped rebar in the gap area changes greatly due to the temperature gradient on the basis of the structural self-weight. Under the action of a positive temperature gradient, the stress increase in the first group of door-shaped rebar is the largest, which is 225.0%. Because the deformation state of the track structure under the positive temperature gradient is the upward arch in the track slab, the fifth group of door-shaped rebar changes from tension to compression. Under the action of a negative temperature gradient, due to the warping deformation of the track slab corner, the interface of the first to fourth groups of door-shaped rebar is converted into tensile stress, and the fifth group of door-shaped rebar is still compressive stress.

### 5.2. Stress Distribution of Door-Shaped Rebar Under the Gap Under the Rail

**Figure 19:**
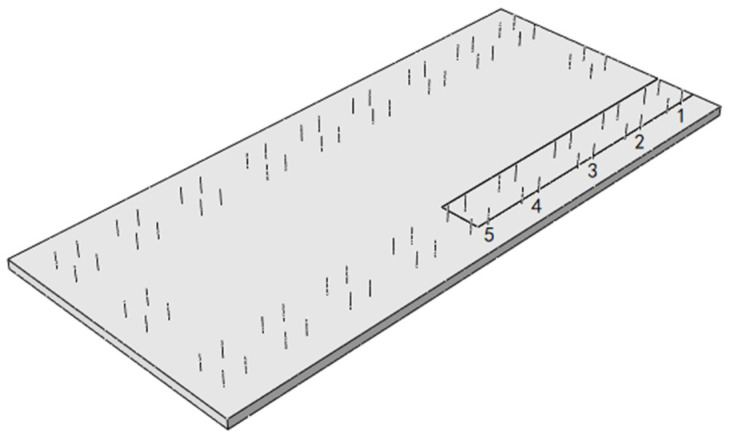
SCC gap under the rail.

The gap under the rail is taken from the SCC layer with a gap width of 400 mm and a height of 2 mm. The gap starts from the position under the rail at the end of the slab and develops longitudinally along the line to the middle of the slab. The relative position of the gap area and the door-shaped rebar is shown in Figure 19. It can be seen from the figure that there are five groups of door-shaped rebars located within the gap range, which are numbered 1–5 from the end of the track slab to the middle of the track slab, and the stress distribution of the door-shaped rebar in this state is analyzed based on this.

**Figure 20:**
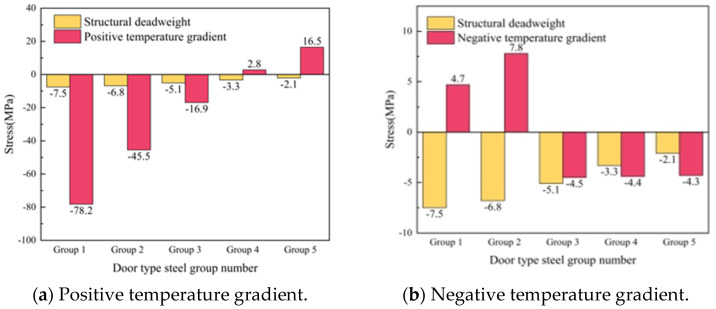
Stress of the door-shaped rebar in the gap area under the rail.

After the gap appears under the rail, the stress of the door-shaped rebar unit in the gap area changes significantly under the action of the track structure’s deadweight and temperature load. The stresses of the five groups of door-shaped rebars at the gap interface under the action of deadweight and after the application of positive and negative temperature loads are shown in Figure 20. Under the action of the deadweight of the structure, the compressive stress of the door-shaped rebar in the gap area increases. The stress value of the door-shaped rebar in the first group (slab end position) is the largest, which is 7.5 MPa. It gradually decreases along the longitudinal direction of the line toward the middle of the slab. The stress of the door-shaped rebar in the fifth group (slab middle position) is 2.1 MPa, which is 28.0% of the maximum value, which is relatively small compared with the gap stress value at the edge of the slab. The stress of the door-shaped rebar in the gap area changes greatly due to the temperature gradient on the basis of the structural self-weight. Under the action of a positive temperature gradient, the stress increase in the first group of door-shaped rebar is the largest, which is 1042.6%. The stress value of the door-shaped rebar decreases along the longitudinal direction toward the middle of the track slab. Because the deformation state of the track structure under the action of the positive temperature gradient is the upward arch in the track slab, when it comes to the fourth and fifth groups of door-shaped rebars, the vertical stress at the gap area changes from tension to compression, and the maximum tensile stress is 16.5 MPa. Under the action of a negative temperature gradient, due to the warping deformation of the track slab corners, the stress at the interface of the first and second groups of door-shaped rebars is converted into tensile stress, with the maximum tensile stress value being 7.8 MPa. The third to fifth groups of door-shaped rebar are still compressive stress.

### 5.3. Stress Distribution of Door-Shaped Rebar Under the Track Slab Middle Gap

**Figure 21:**
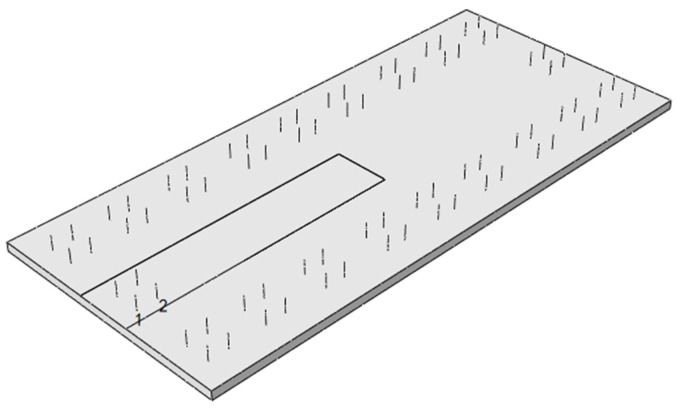
SCC slab middle gap.

The gap in the middle of the slab is taken from the SCC layer with a gap width of 500 mm and a height of 2 mm. The gap develops from the position between the two rails at the end of the slab along the longitudinal direction of the line to the middle of the slab. The relative position of the gap and the door-shaped rebar is shown in Figure 21. It can be seen from the figure that only one group of door-shaped rebar is located within the gap range. This group of door-shaped rebar is numbered 1 and 2 from the end of the track slab to the middle of the track slab, and the stress distribution of the door-shaped rebar in this state is analyzed based on this.

**Figure 22:**
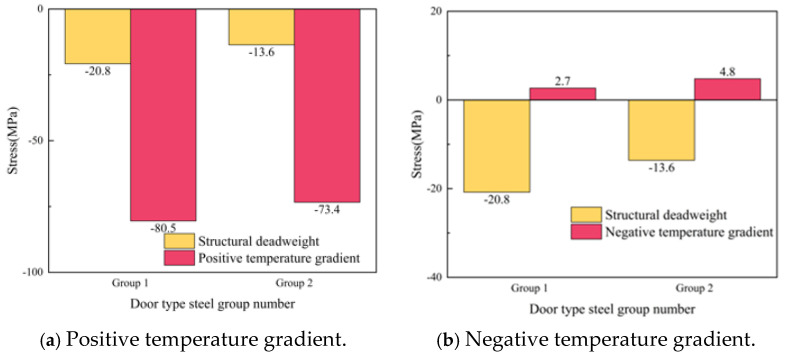
Stress of the door-shaped rebar in the track slab middle separation area.

After the slab is separated, the stress of the door-shaped rebar unit in the separation area changes significantly under the action of the deadweight of the track structure and the temperature load. The stress at the interface of the door-shaped rebar under the action of deadweight and after the application of positive and negative temperature loads is shown in Figure 22. Under the action of the deadweight of the structure, the compressive stress of the door-shaped rebar in the gap area increased. The stresses of the two door-shaped rebars in the gap area were 20.8 MPa and 13.3 MPa, respectively. On the basis of the deadweight of the structure, the door-shaped rebars in the gap area were affected by the temperature gradient, and their stresses changed greatly. Under the action of a positive temperature gradient, the stress difference between the two door-shaped rebars is not significant, which are 80.5 MPa and 73.4 MPa, respectively; under the action of a negative temperature gradient, the stress at the interface of the two door-shaped rebars is converted into tensile stress, which are 2.7 MPa and 4.8 MPa, respectively.

### 5.4. Stress Distribution of Door-Shaped Rebar Under Complete Interface Separation

**Figure 23:**
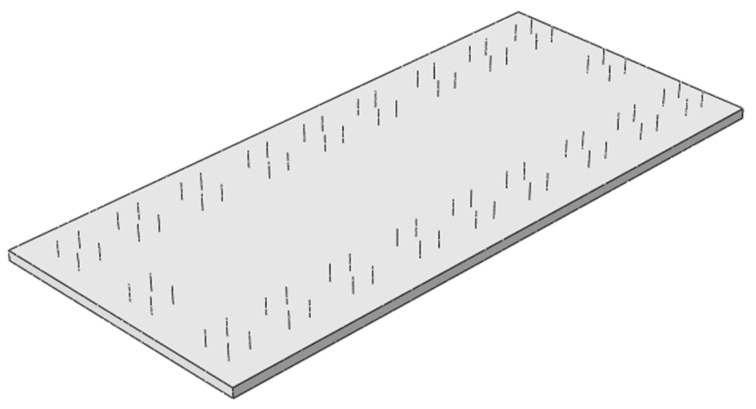
SCC interface is completely separated.

The interface complete separation occurs when a complete separation occurs between the SCC layer and the track slab. The entire interface is connected only by door-shaped rebar. The separation height is 2 mm. The relative positions of the separation and door-shaped rebar are shown in Figure 23. It can be seen from the figure that all the door-shaped rebars are exposed within the gap range. According to the symmetry of the structure, five groups of door-shaped rebars from the track slab end to the track slab middle are analyzed and numbered as groups 1–5 in sequence. The stress distribution of the door-shaped rebar under this state is analyzed accordingly.

**Figure 24:**
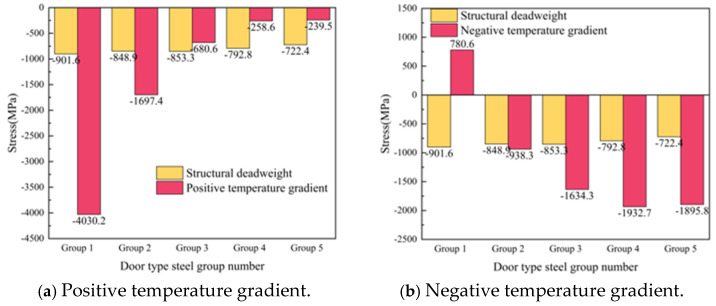
Stress of the door-shaped rebar in the fully separated area.

Complete interface separation is an extreme condition of interface damage. The stress of the door-shaped rebar unit in the separation area changes significantly under the action of the deadweight of the track structure and temperature load. The stresses of the five groups of door-shaped rebar interfaces under the action of deadweight and after the application of positive and negative temperature loads are shown in Figure 24. Under the action of the deadweight of the structure, since the door-shaped rebars need to bear the deadweight of the entire track structure, the door-shaped rebars in the gap area are subjected to significant compressive stress. The stress value of the door-shaped rebar in the first group (slab corner position) is the largest, which is 901.6 MPa. It decreases slightly along the longitudinal direction of the line toward the middle of the slab. The stress of the door-shaped rebar in the fifth group (slab middle position) is 722.4 MPa, which is 80.1% of the maximum value. The stress of the door-shaped rebar in the gap area changes greatly due to the temperature gradient on the basis of the structural self-weight. Under the positive temperature gradient, the stress of the first group of door-shaped rebar increases sharply, with an increase of 347.2%. Because the deformation state of the track structure under the positive temperature gradient is the camber in the track slab, starting from the third group of door-shaped rebar, the stress decreases on the basis of the deadweight, and the minimum compressive stress is obtained at the fifth group of door-shaped rebar, which is 239.5 MPa. Under the action of negative temperature gradient, the first group of door-shaped rebars was transformed into tensile stress at the interface with a stress value of 780.6 MPa, while the second to fifth groups of door-shaped rebars were still compressive stress.

### 5.5. Stress Distribution Pattern of Door-Shaped Rebar Under Different Interface Gap Positions

**Figure 25:**
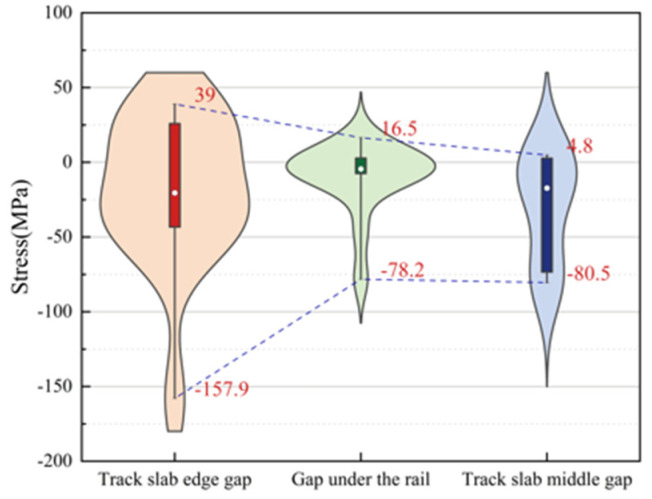
Stress distribution of door-shaped rebar under different interface gap positions.

Figure 25 is a violin plot with box plots reflecting the stress distribution of door-shaped rebar under various joint separation conditions at different interface positions. The maximum compressive stresses of the door-shaped rebar under conditions of the track slab edge gap, gap under the rail and track slab middle gap are 157.9 MPa, 78.2 MPa and 80.5 MPa, respectively, while the maximum tensile stresses are 39 MPa, 16.5 MPa and 4.8 MPa, respectively. It is evident that the track slab edge gap has the greatest impact on the stress of the door-shaped rebar. Additionally, under the track slab edge gap condition, the upper and lower sections of the violin plot are noticeably wider, whereas the width is greatest in the middle for the gap under the rail. This indicates that higher stress values are more densely distributed under the track slab edge gap condition, while stress values for the gap under the rail are concentrated around the median. This confirms that the track slab edge gap is a less favorable condition, and it warrants particular attention in engineering practice.

## 6. Conclusions

Based on the finite element model, this paper analyzes and studies the mechanical characteristics of door-shaped rebar under the maximum positive and negative temperature gradients when the SCC layer is defective and the gap is working. The main conclusions are as follows:

(1) Under the action of the temperature load, the maximum vertical displacement of the track slab occurs in the center and corner positions of the slab under a positive temperature gradient, with displacement values of 0.87 mm and −0.99 mm, respectively; under positive and negative temperature gradients, the stress of the door-shaped rebar at the interface between the track slab and the SCC layer is 12.9 MPa and 16.5 MPa, respectively.
(2) When considering the conditions of the SCC slurry layer, the overall stress increase of the door-shaped rebar in the SCC layer under the action of the temperature gradient was not significant, with the increases of positive temperature gradients being 15.1% and 9.7% and those of negative temperature gradients being 2.0% and 1.6%, respectively.
(3) When the interface of the SCC layer is separated, the stress of the door-shaped rebar unit located in the separation area changes significantly under the action of the deadweight of the track structure and the temperature load. Among them, the separation of the track slab edge has the greatest impact on the stress of the door-shaped rebar under the positive temperature gradient, which is 157.9 MPa. In engineering practice, monitoring and repair measures should be taken to focus on preventing and controlling this fault.
(4) In future work, we will consider the coupling of more factors, because as the track structure accumulates in use time, more problems will be exposed, such as the impact of rainwater.

## Figures and Tables

**Table 1:** Material parameters of each component of the model.

Part	Elastic Modulus MPa	Density kg/m^3^	Poisson’s Ratio	Coefficient of Thermal Expansion /°C
Rail	2.06 × 10^5^	7.80 × 10^3^	0.3	1.18 × 10^−5^
Track Slab	3.65 × 10^4^	2.50 × 10^3^	0.2	1.0 × 10^−5^
SCC Layer	3.40 × 10^4^	2.40 × 10^3^	0.2	1.0 × 10^−5^
Support Layer	3.20 × 10^4^	2.50 × 10^3^	0.2	1.0 × 10^−5^
Surface Layer of Subgrade	300	1.95 × 10^3^	0.3	/
Bottom Layer of Subgrade	250	1.90 × 10^3^	0.25	/

## Data Availability

The original contributions presented in this study are included in the article material. Further inquiries can be directed to the corresponding author(s).
